# Time-restricted eating and age-related muscle loss

**DOI:** 10.18632/aging.102384

**Published:** 2019-10-20

**Authors:** Grant M. Tinsley, Antonio Paoli

**Affiliations:** 1 Department of Kinesiology and Sport Management, Texas Tech University, Lubbock, TX 79409, USA; 2 Department of Biomedical Sciences, University of Padova, Padova, Italy

**Keywords:** intermittent fasting, time-restricted feeding, sarcopenia, skeletal muscle, protein

In recent years, the potential benefits of incorporating regular periods of fasting have been explored for improving health and combating disease [1,2]. While numerous rodent studies have demonstrated potential benefits of fasting for prevention or treatment of age-related diseases, including diabetes, cardiovascular disease, neurological disorders, and cancer, more limited evidence is available in humans [1]. Although preliminary findings indicate that the incorporation of fasting may hold promise for some of the aforementioned conditions, whether fasting would be beneficial, inconsequential, or deleterious is less clear for others. For example, sarcopenia - the loss of skeletal muscle mass and function associated with old age - is a notable concern of the aging population. The functional limitations and physical disability attendant to sarcopenia contribute to reduced quality of life and mortality [3].

The primary anabolic stimuli that can help preserve or increase skeletal muscle mass with aging are exercise and protein intake [4]. Based on investigations of acute muscle protein synthesis responses, it has been recommended to evenly distribute protein intake over the course of waking hours and to consume per-meal protein doses that maximize muscle protein synthetic responses (~0.4 g/kg/meal, with a daily intake of ~1.2 g/kg/d) [4]. The even distribution of protein throughout the day may be viewed as generally at odds with programs incorporating daily fasting periods longer than an overnight fast, such as time-restricted eating (TRE; also known as time-restricted feeding) in which all calories are consumed within a truncated period of time each day. However, some TRE programs would be amenable to the general recommendation of consuming protein boluses of ≥ 0.4 g/kg/meal for ≥2 meals per day. For example, the commonly employed 8-hour eating window could allow for 2 to 3 meals with protein doses ≥ 0.4 g/kg/meal and a daily intake of ≥ 1.2 g/kg, with a relatively minor consolidation of eating occasions relative to longer eating windows of ≥ 10 hours. Additionally, as it has been noted that the elderly experience a diminished anabolic response to a given quantity of protein as compared to young adults [4], there is a possibility that consolidating more numerous eating occasions into several larger boluses could actually aid in meeting per-meal protein intakes necessary for maximal stimulation of muscle protein synthesis. Furthermore, protein intake can be manipulated independent of eating frequency such that a suitable per-meal and total protein intake is achieved with substantially different eating schedules. Nonetheless, the potential importance of missed eating opportunities with TRE, particularly with reference to dietary protein requirements to combat age-related muscle loss, are worthy of consideration.

Optimal strategies for combatting sarcopenia likely involve both nutritional and physical activity or exercise components when functional limitations do not preclude the latter. While data demonstrating the influence of fasting programs on muscular outcomes in active, aging humans are generally unavailable, we have reported such effects in younger adults performing resistance exercise training [5–7]. While an apparent attenuation of lean soft tissue accretion with TRE was observed in one investigation [5], without attendant compromises of muscular performance improvements, the self-selection of a lower protein intake due to the substantially truncated 4-hour eating window likely contributed to this result. Both of our trials employing daily TRE with an ~8-hour eating window have demonstrated equivalent lean mass and skeletal muscle changes in TRE and control groups when both groups consumed 1.6 to 1.9 g/kg/d of protein and similar total energy [6,7]. Most recently, we reported equivalent lean mass accretion and increases in skeletal muscle thickness with daily TRE, in which all calories were consumed within ~7.5 h/day on average, as compared to a control group which ate over the course of ~13 h/day on average [6].

Aging leads not only to a reduction of muscle mass but also to a reduction of specific strength (i.e. the ratio between muscular strength and cross-sectional area), which may be associated with changes in autophagosome formation and autophagosome-lysosome fusion [8]. Indeed, dysfunctional autophagy processes, resulting in impaired removal of damaged organelles and proteins, may lead to decrements in muscle function. Thus, an intriguing hypothesis worthy of future investigation is that periods of fasting could potentially benefit muscle function and efficiency in the elderly through their effects on autophagy machinery.

In summary, nutritional programs incorporating regular periods of fasting may hold promise for combatting some age-related conditions. However, fasting periods may appear at odds with recommended interventions for other conditions, such as sarcopenia. While the specific fasting program in question dictates the extent of this incongruity, some programs, such as TRE, could be employed while still allowing for adequate per-meal and daily protein intake. Although our preliminary investigations of TRE in young adults demonstrate no inherently untoward effects of TRE combined with resistance exercise training, provided that protein and calorie intake are sufficient, it is possible that these results would not be replicated in the elderly. Conversely, it is possible that fasting-induced molecular changes in skeletal muscle could benefit aspects of muscle function in aging populations. A consideration of the potential risks and benefits of these fasting programs for the elderly should be considered. There is a continued need for clinical research to identify optimal lifestyle strategies to combat age-related disease, promote longevity, and improve quality of life in aging adults. Consensus on these strategies has not yet been reached, and it has previously been suggested that implementation of optimal strategies could take decades after consensus is achieved [2].

## References

[1] Mattson MP, et al. Ageing Res Rev. 2017; 39:46–58. 10.1016/j.arr.2016.10.005 27810402 PMC5411330

[2] Longo VD, Panda S. Cell Metab. 2016; 23:1048–59. 10.1016/j.cmet.2016.06.001 27304506 PMC5388543

[3] Cruz-Jentoft AJ, et al. Age Ageing. 2010; 39:412–23. 10.1093/ageing/afq034 20392703 PMC2886201

[4] Traylor DA, et al. Adv Nutr. 2018; 9:171–82. 10.1093/advances/nmy003 29635313 PMC5952928

[5] Tinsley GM, et al. Eur J Sport Sci. 2017; 17:200–07. 10.1080/17461391.2016.1223173 27550719

[6] Tinsley GM, et al. Am J Clin Nutr. 2019; 110:628–40. 10.1093/ajcn/nqz126 31268131 PMC6735806

[7] Moro T, et al. J Transl Med. 2016; 14:290. 10.1186/s12967-016-1044-0 27737674 PMC5064803

[8] Aas SN, et al. Exp Gerontol. 2019; 125:110687. 10.1016/j.exger.2019.110687 31404624

